# Multi-Fundus Diseases Classification Using Retinal Optical Coherence Tomography Images with Swin Transformer V2

**DOI:** 10.3390/jimaging9100203

**Published:** 2023-09-29

**Authors:** Zhenwei Li, Yanqi Han, Xiaoli Yang

**Affiliations:** College of Medical Technology and Engineering, Henan University of Science and Technology, Luoyang 471023, China; 210321221641@stu.haust.edu.cn (Y.H.); yxl@haust.edu.cn (X.Y.)

**Keywords:** multi-fundus diseases classification, optical coherence tomography, Swin Transformer V2, PolyLoss function, OCT2017 and OCT-C8

## Abstract

Fundus diseases cause damage to any part of the retina. Untreated fundus diseases can lead to severe vision loss and even blindness. Analyzing optical coherence tomography (OCT) images using deep learning methods can provide early screening and diagnosis of fundus diseases. In this paper, a deep learning model based on Swin Transformer V2 was proposed to diagnose fundus diseases rapidly and accurately. In this method, calculating self-attention within local windows was used to reduce computational complexity and improve its classification efficiency. Meanwhile, the PolyLoss function was introduced to further improve the model’s accuracy, and heat maps were generated to visualize the predictions of the model. Two independent public datasets, OCT 2017 and OCT-C8, were applied to train the model and evaluate its performance, respectively. The results showed that the proposed model achieved an average accuracy of 99.9% on OCT 2017 and 99.5% on OCT-C8, performing well in the automatic classification of multi-fundus diseases using retinal OCT images.

## 1. Introduction

Fundus diseases include conditions such as diabetic macular edema (DME), choroidal neovascularization (CNV), and drusen, which significantly impact the quality of life [1]. With the continuous development of ophthalmic medicine, OCT technology has become an important diagnostic tool, especially in the diagnosis of fundus diseases. OCT is a non-invasive imaging technique that provides high-resolution retinal images to help diagnose eye diseases, evaluate treatment outcomes, and monitor disease progression [2]. However, due to the large amount of data and complex structural and morphological features of retinal OCT images, manual diagnosis requires a significant amount of time and effort. Therefore, computer-aided diagnosis (CAD) techniques have significant value in the automatic classification of retinal OCT images.

CAD refers to the use of computer technology to analyze and process medical images to provide diagnostic assistance [3]. CAD is now widely used in the automatic analysis and diagnosis of medical images, such as breast cancer, lung cancer, and colorectal cancer. CAD systems can help doctors diagnose diseases quickly and accurately, improving diagnostic accuracy and efficiency. Deep learning is a machine learning technique that has been widely applied in the field of computer-aided diagnosis [4]. Convolutional neural networks (CNNs) are a type of deep learning technique that has been continuously developed since the 1980s. CNNs have achieved great success in the field of computer vision and are widely used in tasks such as image classification, object detection, and semantic segmentation. Some early CNN models include LeNet [5] and AlexNet [6]. As deep learning technology has continued to develop, many new CNN models have emerged, including VGGNet [7], GoogLeNet [8], ResNet [9], DenseNet [10], MobileNet [11], and EfficientNet [12]. Although the existing models have achieved great success, there is still room for improvement in the classification of fundus diseases using OCT images.

Unlike RNNs, which require recursive processing to obtain global information, or CNNs, which can only obtain local information, Transformer is a new neural network architecture that can directly obtain global information. Transformer is essentially an Attention structure that can perform parallel computations, and is therefore much faster than RNNs. Transformer network architecture was proposed by Ashish Vaswani et al. in their paper “Attention Is All You Need” and has been used for machine translation tasks. Unlike previous network architectures, the encoder and decoder in this architecture do not use RNN or CNN network architectures, but instead rely on an architecture that is completely dependent on the attention mechanism [13].

The Swin Transformer is a novel Transformer model that has achieved excellent performance in many computer vision tasks [14]. Compared to the traditional Vision Transformer (ViT) [15], the Swin Transformer utilizes a multi-scale design and integrates the multi-scale design into the Transformer. One of the main features of the Swin Transformer is its pyramidal structure, i.e., the deeper the network is, the smaller the size of the feature map is, and the more channels are available. This is different from the columnar structure of ViT, where the feature map size remains constant. In addition, the Swin Transformer borrows many techniques from CNNs, such as hierarchical feature extraction (FPN), Sliding Window + Attention Mask + Cyclic Shift. These techniques help the Swin Transformer to better capture local information in the image and extract multi-scale features. In conclusion, by adopting a multi-scale design and borrowing techniques from CNNs, the Swin Transformer achieves better performance than traditional ViT models in several computer vision tasks.

### 1.1. The Proposed Model

In this paper, we propose a multi-foveal disease classification model based on Swin Transformer V2 [16]. The dataset is first subjected to preprocessing operations such as data enhancement, and then the network is trained. Based on the results of training, the network parameters such as learning rate and batch size are fine-tuned to determine the appropriate training parameters. By comparing different loss functions, we finally adopted PolyLoss [17] as the loss function to obtain better performance in retinal OCT image classification. In order to improve the interpretability of the model and understand its decision-making process, visualization methods such as the confusion matrix and Grad-CAM heatmap [18] were used in the testing phase. Finally, after continuous optimization of network parameters and loss functions, the results were compared after multiple training sessions to obtain the optimal network model for multiple fundus disease classification.

The contributions of this paper are as follows:

1. The proposed method will first use the Swin Transformer V2 model to classify multiple diseases in retinal OCT images.
2. Based on the Swin Transformer V2 model, its loss function is improved by introducing PolyLoss, which improves the model’s performance.
3. Experimental validation was performed with two datasets, OCT2017 and OCT-C8, and using Grad-CAM visualization to help understand decision-making mechanisms in network models.

### 1.2. Related Work

The use of deep learning algorithms for identifying OCT images has been extensively studied by many researchers. For example, Lee et al. used a deep neural network to classify OCT images as normal or AMD, achieving an accuracy of 87.63% [19]. Lu et al. and Bhadra et al. used a deep multi-layer CNN to categorize OCT images into healthy, dry AMD, wet AMD, and DME [20]. Kermany et al. applied deep transfer learning to automatically diagnose diabetic retinopathy in OCT images [21]. Rong et al. suggested a different auxiliary classification method, based on CNNs, for the automatic categorization of retinal OCT images [22]. Fang et al. proposed a novel lesion-aware convolutional neural network (LACNN) method for retinal OCT image classification, where retinal lesions in OCT images were used to guide the CNN to achieve more accurate classification [23]. Singh et al. studied attribute-explained deep learning: application to ophthalmic diagnosis and proposed a framework for explaining the classification decisions of a deep learning network on retinal OCT images [24]. Wang et al. proposed classifying volumetric OCT images via a recurrent neural network (VOCT-RNN), which can fully exploit temporal information among B-scans. This choice may introduce unnecessary model complexity, limiting the interpretation of such model results in clinical practice [25]. To investigate this hypothesis, Arefin et al. developed a configurable deep convolutional neural network (CNN) that classifies four types of macular diseases using retinal optical coherence tomography (OCT) images [26]. V et al. proposed a method to improve the automatic classification and detection of macular diseases using retinal optical coherence tomography (OCT) images by fusing two pre-trained deep learning networks [27]. Identifying macular diseases and segmenting lesion areas to assist ophthalmologists in clinical diagnosis is necessary. Liu et al. studied joint disease classification and lesion segmentation in OCT images via a one-stage attention-based convolutional neural network [28]. Deep-learning-based methods have been proposed to address this problem. To evaluate the proposed method, Esfahani et al. used publicly available data including 45 OCT volumes, 15 age-related macular degeneration, 15 diabetic macular edema, and 15 normal volumes captured by Heidelberg OCT imaging equipment [29]. He et al. proposed a method for classifying retinal OCT images using an interpretable Swin-Poly Transformer network [30]. This is a significant contribution to the field of retinal OCT image classification. At the same time, our work has been inspired by this study, and we have improved upon it. Other influential works include those by Lbrahim, Ai, Z, etc. [31,32]. However, to achieve fast and accurate detection results, it is necessary to break out of the existing CNN framework, which is challenging.

The Transformer is a type of model architecture in the field of natural language processing (NLP). Its relatively mature theoretical support and technological development in the field of natural language processing have brought it to the attention of researchers, and it has been shown that Transformer methods can be applied to computer vision tasks, outperforming existing CNN methods in some tasks [33]. The Vision Transformer (ViT) is a model proposed by the Google team in 2020 that applies the Transformer to image classification. Its model is “simple” and effective, with strong scalability (the larger the model, the better the performance), and performs well in the field of computer vision. The Swin Transformer is a new type of visual Transformer that can serve as a general backbone network for computer vision. It adopts a hierarchical structure and shifted windows to effectively extract multi-scale features. In addition, some researchers have attempted to combine Transformers and CNNs to improve prediction performance. For example, when performing object detection in drone images, a Transformer-based model can be fused with a CNN-based model [34]. Swin Transformer V2 is a large model for computer vision that addresses three main issues in training and applying large visual models, including training instability, the resolution gap between pre-training and fine-tuning, and the need for labeled data. Swin Transformer V2 can better handle complex image data and achieve excellent performance in the automatic classification of retinal OCT images.

## 2. Materials and Methods

**Figure 1:**
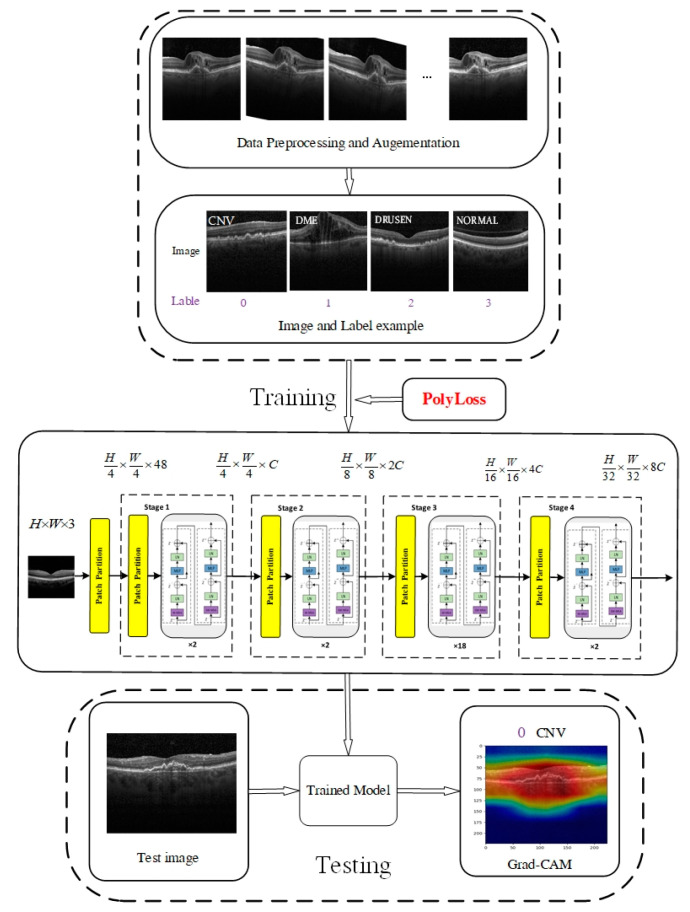
The overall framework of the proposed method.

The overall framework of the proposed method is illustrated in Figure 1. The PolyLoss loss function is employed during the experiment to enhance the training efficiency of the model. Data augmentation methods are applied during the training phase to increase the diversity of the training data and enhance the network’s ability to generalize. After training, Grad-CAM is utilized to visualize and explain the results.

### 2.1. Architecture of Swin Transformer V2

Swin Transformer V2 is an upgraded version of Swin Transformer. It improves upon version 1.0 by making the model larger and able to adapt to different image resolutions and window sizes. The Swin Transformer V2 block incorporates two Swin Transformer modules, the window multi-head self-attention (W-MSA) module and the shifted window multi-head self-attention (SW-MSA) module, in place of the standard multi-head self-attention (MSA) module found in ViT. In addition, when calculating Attention in the Transformer block in ViT, the dot(Q,K) operation is used, which is replaced by cosine(Q,K)/τ in Swin V2, where τ is a learnable parameter that is not shared between blocks. The cosine operation inherently includes normalization, which further stabilizes the attention output values.

**Figure 2:**
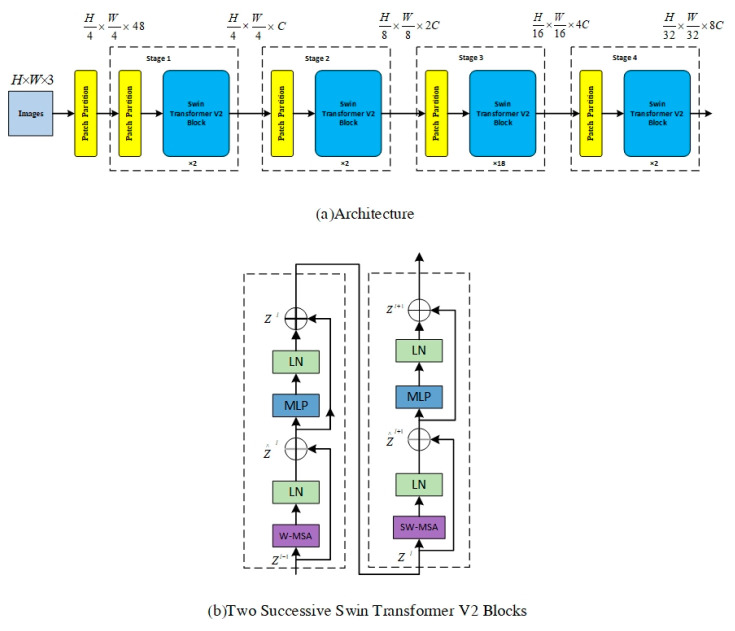
(**a**) he overall architecture of Swin Transformer V2. (**b**) Two successive Swin Transformer V2 blocks.

**Figure 3:**
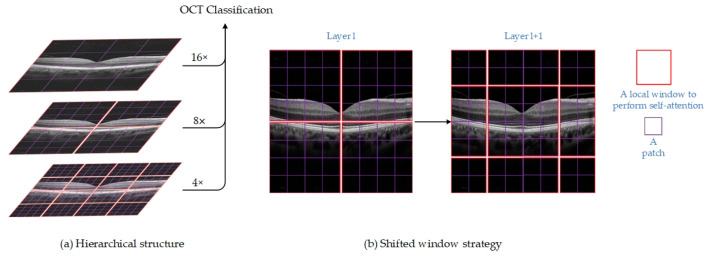
(**a**) The hierarchical structure of Swin Transformer V2 for extracting multi-scale feature representation. (**b**) An illustration of the shifted window strategy for computing self-attention in the Swin Transformer V2 architecture.

Figure 2 illustrates the overall structure of the Swin Transformer V2 model [14]. The input image, with a size of 256 × 256, is first divided into non-overlapping 4 × 4 patches by the patch partitioning module. These patches are then treated as ‘tokens’ and projected into C dimensions using a linear embedding layer. Two consecutive Swin Transformer V2 blocks with self-attention computation are applied to these patch tokens, controlling their number as shown in Figure 2b. A ‘stage’ consists of a linear embedding layer and Swin Transformer V2 blocks. The design of Swin Transformer V2 resembles the layer structure of CNNs, where the resolution is halved, and the number of channels is doubled at each stage. To produce hierarchical representations, the Swin Transformer reduces the number of tokens by merging patch layers, making the network deeper. Figure 3a shows an example of a hierarchical representation. Differing from the 224 × 224 input resolution used by He et al. [30], we employ Swin Transformer V2, which uses a higher resolution of 256 × 256. The advantage of this is that the network has access to more features, and increasing the feature extraction capability of the network improves the performance of the model.

Each Swin Transformer V2 block comprises two units, with each unit containing two normalization layers (LayerNorm), a self-attention module, and a multi-layer perceptron (MLP) layer. The standard multi-head self-attention (MSA) module from ViT is replaced by two consecutive Swin Transformer V2 modules in the Swin Transformer V2 block: the window multi-head self-attention (W-MSA) module and the shifted window multi-head self-attention (SW-MSA) module, as shown in Figure 2b. The first unit utilizes the window MSA (W-MSA) module, while the second unit employs the shifted window MSA (SW-MSA) module. In contrast to the Swin Transformer, Swin Transformer V2 incorporates a LayerNorm layer after each MSA module and MLP layer and implements residual connections after each module.

### 2.2. Shifted-Window-Based Self-Attention

A method of calculating self-attention within local windows is used to reduce computational complexity and improve modeling efficiency. The moving window strategy used to calculate self-attention in this experiment is shown in Figure 3a. In the ViT architecture, the standard MSA module is used for global attention, resulting in an unbearable amount of computation and quadratic computational complexity. In W-MSA, this relationship is linear, and the amount of computation is acceptable. Assuming that each window includes M × M patches, windows are organized in a non-overlapping manner to split the image in an equal amount. On an image with hardware patches, the global MSA module’s computational complexity and the window-based MSA module’s computational complexity are, respectively:

(1) $$\Omega \left(\mathrm{MSA}\right)=4hwC^{2}+2{\left(hw\right)}^{2}C$$

(2) $$\Omega \left(\mathrm{W-MSA}\right)=4hwC^{2}+2M^{2}hwC$$

where h × w is the total number of patches in the picture, and C denotes the patch channel’s channel. When M is constant (the default value is 7), the complexity of Equation (2) is linear as opposed to Equation (1), where the difficulty is quadratic with respect to the number of patches h × w.

**Figure 4:**
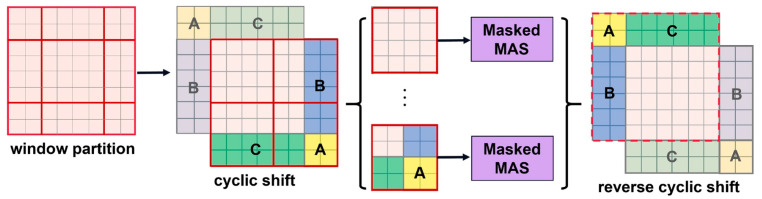
Illustration of an efficient batch computation approach for self-attention in shifted window partitioning.

The window-based self-attention module lacks cross-window connections, ignoring the relationships between different windows and limiting modeling capabilities. This approach switches between two partition configurations in succeeding Swin Transformer V2 blocks to set up cross-window connections while retaining the computational efficiency of non-overlapping windows. As identified in Figure 4 [14], the first module equally divides the 8 × 8 feature map into 2 × 2 windows of size 4 × 4 (M = 4) using a standard window partitioning approach starting from the top-left pixel. Then, the next module adopts a window configuration that is offset from the previous layer’s window configuration by shifting the window from the regular partitioned window by ($\left[\frac{M}{2}\right]$, $\left[\frac{M}{2}\right]$)pixels. In the new window, the self-attention calculation also takes into account the boundary of the previous window, thus considering the connection information between different windows. Using the shifted window partitioning method, consecutive Swin Transformer V2 blocks are calculated as:

(3) $${\hat{Z}}^{l}=\mathrm{W-MSA}(\mathrm{LN}(Z^{l-1}))+Z^{l-1}$$

(4) $$Z^{l}=\mathrm{MLP}(\mathrm{LN}({\hat{Z}}^{l}))+{\hat{Z}}^{l}$$

(5) $${\hat{Z}}^{l+1}=\mathrm{SW-MSA}(\mathrm{LN}({\hat{Z}}^{l}))+{\hat{Z}}^{l}$$

(6) $$Z^{l+1}=\mathrm{MLP}(\mathrm{LN}({\hat{Z}}^{l+1}))+{\hat{Z}}^{l+1}$$

where W-MSA and SW-MSA indicate window-based multi-head self-attention utilizing normal and shifted window partitioning configurations, respectively; and ${\hat{Z}}^{l}\;$and $Z^{l}\;$denote the output characteristics of the (S)W-MSA module and MLP in the l layer, respectively.

A number of new windows are produced by the window partitioning technique, some of which are smaller than M × M. One typical method for calculating self-attention is to flatten all windows to M × M. This method, however, results in more windows. For instance, in Figure 3b, the window transformation technique results in a large rise in the computational cost of the model when the number of windows goes from 2 × 2 to 3 × 3. As demonstrated in Figure 4, we apply an effective batch computation technique that cyclically shifts to the top left to address this problem. The batch-calculated windows may include a number of non-adjacent windows in the feature map after shifting. Therefore, to confuse the self-attention calculation for each sub-window, we use a masking method. The computational efficiency is increased for cyclic shifting since the number of batch windows and regular window divisions stays constant.

### 2.3. PolyLoss

The PolyLoss function has been demonstrated to outperform cross-entropy loss and focal loss in tasks such as 3D detection, 2D picture classification, instance segmentation, and object identification. As a result, in this experiment, we adopted PolyLoss as the loss function for our model to improve the OCT classification model’s classification accuracy. The coefficients of the polynomial are represented by, and the PolyLoss formula is expressed as follows:

(7) $$L_{Poly}=\alpha_{1}\left(1-P_{t}\right)+\alpha_{2}{\left(1-P_{t}\right)}^{2}+\cdots +\alpha_{N}{\left(1-P_{t}\right)}^{N}+\cdots =\sum_{j=1}^{\infty }\alpha_{j}{\left(1-P_{t}\right)}^{j}$$

There are an endless number of polynomial coefficients that need to be changed in this formula. Tuning multiple polynomial coefficients would still result in a dauntingly large search space, which is not feasible. Additionally, cross-entropy loss does not perform better than many coefficients being tuned simultaneously. This problem is solved by perturbing the leading polynomial coefficient in the cross-entropy loss while leaving the other coefficients constant. The loss formula is written as Poly-N, where N is the quantity of leading coefficients that need to be changed.

(8) $$L_{Poly-N}=\underset{perturbed\;by\;\varepsilon_{j}}{\underbrace{\left(\varepsilon_{1}+1\right)\left(1-P_{t}\right)+\cdots +\left(\varepsilon_{N}+1/N\right){\left(1-P_{t}\right)}^{N}}}+\underset{same\;as\;CrossEntropy}{\underbrace{1/\left(N+1\right){\left(1-P_{t}\right)}^{N+1}+\cdots }}\;=-\log(P_{t})+\sum_{j=1}^{N}\varepsilon_{j}{\left(1-P_{t}\right)}^{j}$$

In particular, we update the cross-entropy loss’s j polynomial coefficient from 1/j to $1/j+\varepsilon_{j}$, where $\varepsilon_{j}$∈[−1⁄j,∞) is the perturbation term. Equation (8) demonstrates how the first N polynomials may be precisely computed without having to worry about an endless number of higher-order (j > N + 1) coefficients. The largest increase is possible for the first polynomial term. The final PolyLoss formula is as follows with further simplification of the Poly-N formula and concentration on Poly-1 evaluation, where only the first polynomial coefficient in the cross-entropy loss is changed:

(9) $$L_{Poly-1}=\left(1+\varepsilon_{1}\right)\left(1-P_{t}\right)+1/2{\left(1-P_{t}\right)}^{2}+\cdots =-\log\left(P_{t}\right)+\varepsilon_{1}\left(1-P_{t}\right)$$

In this experiment, we accomplish OCT image classification using the value of $\varepsilon_{1}$ = 2.

### 2.4. Datasets

**Figure 5:**
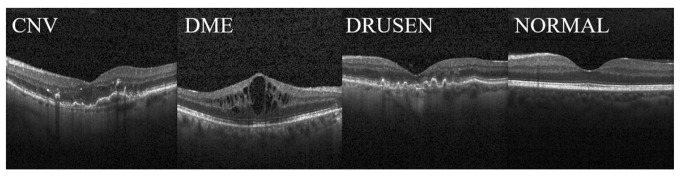
Optical coherence tomography images from the OCT2017 dataset. The panels display images of choroidal neovascularization (CNV) on the far left, diabetic macular edema (DME) on the middle left, drusen on the middle right, and a normal image on the far right.

**Figure 6:**
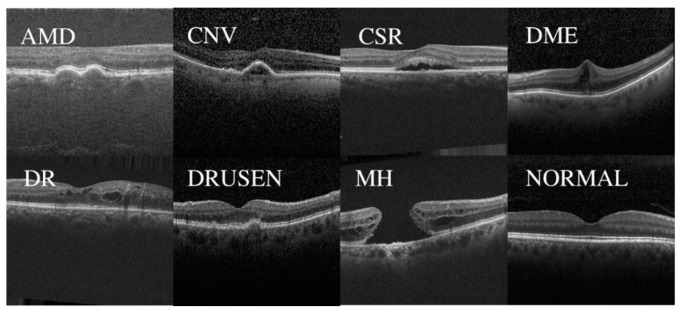
Displays examples of the eight classes in the OCT-C8 dataset, including AMD, CNV, CSR, DME, DR, DRUSEN, MH, and NORMAL.

In this paper, two public datasets, OCT2017 [35] and OCT-C8 [36], were used to train and test the network model. Dataset 1, as shown in Figure 5, depicts examples of three fundus diseases and normal retina, while Dataset 2, as shown in Figure 6 [37], depicts OCT images of seven diseases and one normal category of retinal OCT images. The OCT2017 dataset contains images of three diseases: choroidal neovascularization (CNV), diabetic macular edema (DME), Drusen, and a class of normal fundus. The OCT2017 dataset contains 84,452 retinal OCT images of 4 classes (as shown in Figure 5): 83,484 training images and 968 test images. The training set includes 36,205 CNV images, 10,348 DME images, 7616 DRUSEN images, and 25,315 NORMAL images for training and four classes of 1000 images each for validation. Details of the two datasets have been shown in Table 1.

The OCT-C8 dataset contains 24,000 images of eight categories (as shown in Figure 6), including AMD, choroidal neovascularization (CNV), central serous retinopathy (CSR), DME, diabetic retinopathy (DR), drusen, macular hole (MH), and one for healthy classes. The training set consists of 2300 images per category for a total of 18,400 images for training and 2800 images each for testing and validation containing 350 images per category for the network model. Before training the model, we preprocessed and augmented the data. Obtaining a large number of labeled medical images is challenging due to the time-consuming nature of the labeling process and the need for professional medical expertise, which can be costly. To increase the diversity of the training data, data augmentation methods such as random rotation, cropping, and mirroring were used. Additionally, the images were resized to 256 × 256 and normalized to match the model’s input requirements. In the final step, the data were converted into tensors and fed into the model for training. This process helps to enhance the model’s ability to generalize and improve its stability.

### 2.5. Evaluation Metrics

To evaluate the performance of the model in classification, we use Accuracy, Precision, and Recall as evaluation metrics. The formulas for these evaluation metrics are shown below.

(10) $$\mathrm{Accuracy}=\frac{\mathrm{TP}+\mathrm{TN}}{\mathrm{TP}+\mathrm{TN}+\mathrm{FP}+\mathrm{FN}}$$

(11) $$\mathrm{Precision}=\frac{\mathrm{TP}}{\mathrm{TP}+\mathrm{FP}}$$

(12) $$\mathrm{Recall}=\frac{\mathrm{TP}}{\mathrm{TP}+\mathrm{FN}}$$

(13) $$\mathrm{F1-score}=\frac{2\mathrm{TP}}{2\mathrm{TP}+\mathrm{FP}+\mathrm{FN}}$$

The numbers TP, TN, FP, and FN stand for the corresponding amounts of true positives, true negatives, false positives, and false negatives. For OCT classification, TP is defined as the proportion of cases that the model correctly classified as positive, TN as the proportion of cases that the model correctly classified as negative, FP as the proportion of negative samples that the model incorrectly classified as positive, and FN as the proportion of positive cases that the model incorrectly classified as negative.

## 3. Results

In this research, the network was trained and evaluated on a Windows 10 operating system with 64 GB of memory, an NVIDIA 4090 24 GB GPU, a 2 TB solid-state drive, Python 3.7, and PyTorch 1.10.1 + cu102. At the start of each experiment, we imported ImageNet-22K pre-trained models through transfer learning. The input resolution for the EfficientNetV2 is set to 384 × 384, the VIT and Swin Transformer models are set to 224 × 224, and the V2 model supports higher resolution image input than the Swin Transformer, set to 256 × 256. The batch size was set to 32 and each model was trained for 200 epochs. During training, we saved the models with the highest accuracy and lowest loss function and selected the model with the highest test accuracy as the optimal model through comparison.

The performance of each category in the OCT2017 dataset was tested using pre-trained EfficientNetV2 [38], Vision Transformer (VIT), Swin Transformer, and our improved Swin Transformer V2 network. Table 2 shows the experimental results for the three retinal disease and normal category diagnoses when the CrossEntropy loss function is used for the four network models on the dataset OCT2017. Table 3 shows the experimental results obtained for different network models on the same dataset when using the PolyLoss function.

To further validate our models, we also tested and analyzed the performance of the VIT, Swin Transformer, and Swin Transformer V2 network models on the OCT-C8 dataset using CrossEntropyLoss, with the results shown in Table 4, and the PolyLoss loss function, with the results shown in Table 5, to categorize the performance of the VIT, Swin Transformer, and Swin Transformer V2 network models.

**Figure 7:**
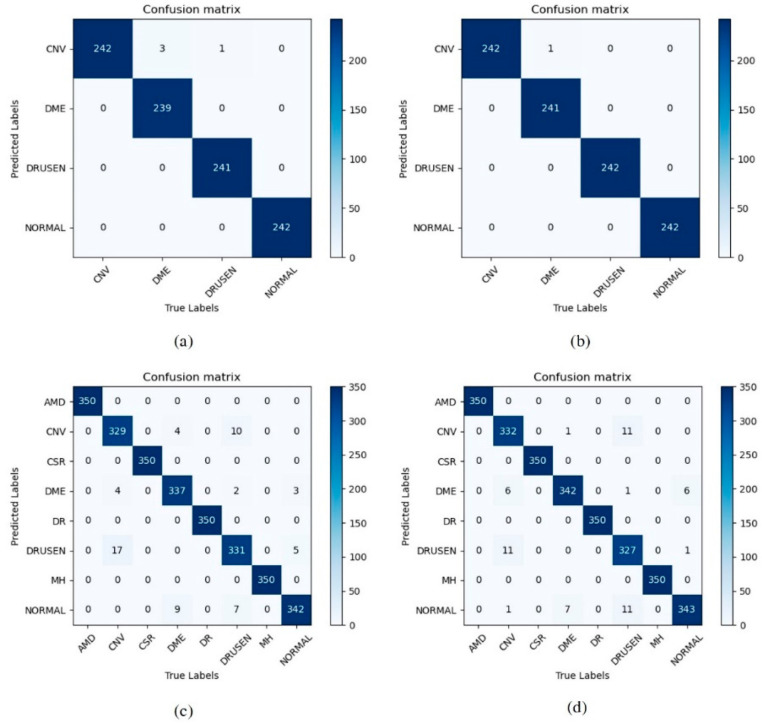
The Confusion matrix of our model on (**a**) OCT2017 (CrossEntropyLoss), (**b**) OCT2017 (PolyLoss), (**c**) OCT-C8 (CrossEntropyLoss), and (**d**) OCT-C8 (PolyLoss).

In order to visualize the performance of each model more intuitively, we use the confusion matrix to visualize the matching results between the model predictions and the true categories. The results obtained by our models on the OCT2017 and OCT-C8 datasets using different loss functions, respectively, are shown in Figure 7a,c are the results when CrossEntropy is applied, and Figure 7b,d represent the results obtained by the PolyLoss function. The diagonal elements in the confusion matrix represent the correct classification, and the remaining elements represent the misclassification.

## 4. Discussion

As can be seen from Table 2, EfficientNetV2 achieved an accuracy of 0.975 in the CNV category, and the highest accuracy of 0.988 was obtained in the normal category, with an F1-Score of 0.953 and 0.976 in the CNV and normal, respectively. The category accuracies of 0.986 and 0.977 were achieved in the DME and DRUSEN, respectively, while the VIT model obtained an overall lower evaluation metric than EfficientNetV2 on all four categories. Both Swin Transformer and our model achieved more than 99% accuracy on a single category, and the evaluation metrics achieved a score of 1 on the normal category. Table 3 shows that when using the PolyLoss function, EfficientNetV2 shows a slight decrease in diagnostic performance on the CNV and DRUSEN categories and a slight increase on the DME and NORMAL categories. The evaluation metrics for the three retinal disease diagnoses improved on Swin Transformer and our model. Compared to the Swin Transformer, our model obtained a higher performance evaluation with a category diagnostic accuracy of 0.999 for both CNV and DME. An accuracy score of 1 was obtained on DEUSEN and normal fundus.

Table 6 is the average of the experimental results obtained using the CrossEntropy and PolyLoss functions on the OCT2017 and OCT-C8 datasets, respectively. We observed that the performance of the EfficientNetV2 network was better than that of VIT when using CrossEntropy loss, with average accuracies of 98.2% and 96.5%, respectively. However, the Swin Transformer model achieved a 3.3% average accuracy improvement over EfficientNetV2 and performed better. We achieved an average accuracy of 99.8% using Swin Transformer V2, which improved on Precision, Recall, Specificity, and F1-Score compared to the Swin Transformer. When the loss function was changed from CrossEntropyLoss to Polyloss, although the Swin Transformer network achieved the same accuracy, it improved in several other evaluation metrics. It can be seen that when using PolyLoss, compared with CrossEntropyLoss, Swin Transformer V2 showed an improvement in Performance, with a 0.3% increase in Precision, a 0.4% increase in Recall, and a 0.1% increase in F1-Score. Swin Transformer V2 achieved 100% Precision, Recall, and Sensitivity in the DME, DRUSEN, and NORMAL categories and achieved near 1.0 accuracy in the CNV, DME, DRUSEN, and NORMAL categories. This proves the excellent classification ability of Swin Transformer V2 on the OCT dataset and that using the PolyLoss loss function can further improve the performance of the network.

On the OCT-C8 dataset, this method outperformed VIT and Swin Transformer, and using the PolyLoss loss function further improved performance, resulting in the best average performance. After using the PolyLoss loss function, Swin Transformer and our Swin Transformer V2 achieved 100% accuracy in the ADM, CSR, DR, and MH categories. In summary, in our experiments, Swin Transformer V2 demonstrated excellent classification ability on the OCT dataset. In addition, we found that using the PolyLoss loss function can further improve the performance of the network.

In addition, we compared our results with other studies. Table 7 shows the results of our comparison. Through comparison, we found that our Swin Transformer V2 improved with PolyLoss, achieving better accuracy and sensitivity performance. This demonstrates the reliability of our method in OCT image classification. These results indicate that our method has high reliability and accuracy in OCT image classification. Our Swin Transformer V2 improved with PolyLoss not only performs well in terms of accuracy, but also achieves good results in terms of sensitivity. These achievements provide strong support for our research in the field of OCT image classification and lay a solid foundation for future research.

**Figure 8:**
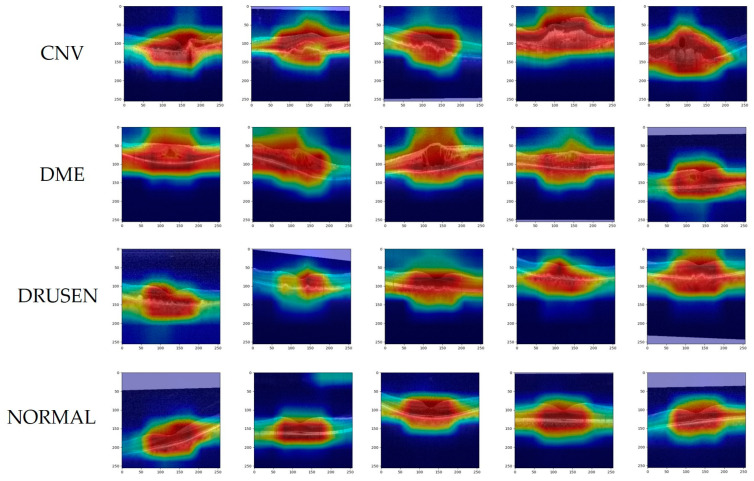
Gradient-weight class activation mapping on OCT2017 of our proposed networks.

Figure 7a,b are the confusion matrices of Swin Transformer V2 using CrossEntropyLoss and PolyLoss when tested with 968 images in the OCT2017 dataset, respectively. Figure 8b represents that the model judged a DME image as CNV disease, while it made zero errors in other categories, thus proving the excellent classification ability of the network. Figure 7c,d are the confusion matrices using two loss functions on 2800 test images in OCT-C8, respectively. As can be seen, the network has successfully classified AMD, CSR, DR, and MH data.

**Figure 9:**
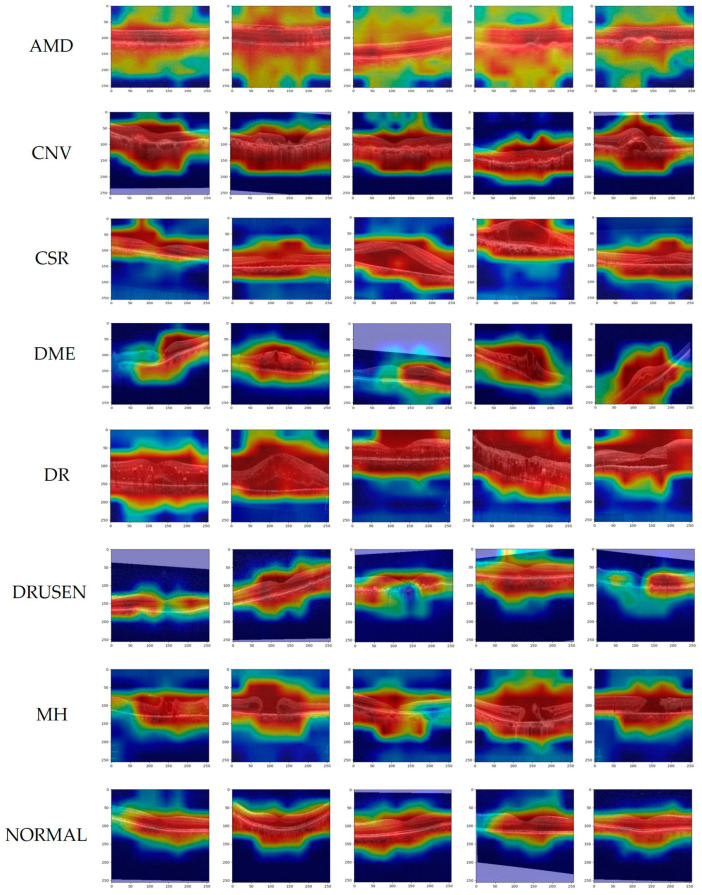
Gradient-weight class activation mapping on OCT-C8 of our proposed networks.

For the trained OCT model, we use Grad-CAM to visualize the decision-making mechanism of the prediction. Grad-CAM is a gradient-based deep network visualization method that explains the classification basis of deep neural network models in the form of heat maps, making category judgments through the pixels of the image. Figure 8 and Figure 9 show heatmaps of the prediction results for the OCT2017 and OCT-C8 datasets, respectively. The colors of the heatmap represent regions of interest, with red indicating high correlation with the target category and blue indicating less attention to the region. The purple area is the result of filling the blank area after data enhancement of the image. Meanwhile, lesion regions show up as a darker red color in disease OCT images. As shown in Figure 8, the second row of images shows the Grad-CAM of the DME image, and from the third image, it can be observed that the region of susceptibility contains the macular edema lesion. The image in the third row and fourth column of Figure 8 shows the region of interest for Drusen and also the region where the lesion occurred. Figure 9 is a partial image of the heat maps of the eight disease categories on the OCT-C8 dataset, showing the prediction of the heat maps of the lesion regions of each disease by our trained model. Grad-CAM helps us to see the regions of interest that the model focuses on when making a prediction, and thus to understand the decision-making process of the prediction. It is worth noting that this focus on the region of interest is also consistent with the ophthalmologist’s observation and diagnostic process.

## 5. Conclusions

In this paper, a multi-fundus disease classification model based on Swin Transformer V2 and the PolyLoss loss function was proposed. By comparing two different loss functions, it has been demonstrated that the PolyLoss function can enhance the model’s functionality. In the final experiment, an evaluation index close to 1 was achieved on the OCT2017 dataset, proving the good performance of the model in classifying OCT images. To validate the generalization ability of the network, it was trained and evaluated on OCT-C8, attaining a score of 1 for accuracy and other assessment metrics in half of the OCT illness categories and an average accuracy of 99.5% on the OCT-C8 dataset, proving the effectiveness of our designed model in classifying fundus diseases on OCT images.

The basic Swin Transformer V2 demonstrated strong performance on the publicly available OCT2017 dataset, making further improvements challenging. In clinical practice, misdiagnosis and missed diagnosis can lead to serious medical accidents and cause great pain to patients. The aim of our work is to improve the accuracy of model automatic diagnosis as much as possible to reduce the occurrence of misdiagnosis and missed diagnosis. However, by using polynomial loss and optimizing the network parameters, we were able to achieve a comprehensive improvement in performance metrics at a high-performance level of 99.7%, achieving a score close to 1. This indicates that our modified network model exhibits superior diagnostic capabilities. Although the magnitude of improvement is relatively small, it has positive implications for reducing misdiagnosis and improving diagnosis.

However, despite the good progress made by deep learning models in identifying abnormalities on retinal OCT images, due to the limited dataset, it is not possible to verify how well they perform on other retinal OCT data. In the future, more retinal OCT data will be sought to validate and improve the network. In addition, turning a network model into a powerful tool in the hands of clinical ophthalmologists in real life is also a major challenge, requiring more professionals to work together to turn theoretical methods into products that improve ophthalmic diagnosis.

## Figures and Tables

**Table 1:** Classification and dataset setup for datasets OCT2017 and OCT-C8.

Dataset	Class	Number	Train	Validation	Test
OCT2017	CNV	37,447	36,205	1000	242
	DME	11,590	10,348	1000	242
	DRUSEN	8858	7616	1000	242
	NORMAL	26,557	25,315	1000	242
OCT-C8	AMD	3000	2300	350	350
	CNV	3000	2300	350	350
	CSR	3000	2300	350	350
	DME	3000	2300	350	350
	DR	3000	2300	350	350
	DRUSEN	3000	2300	350	350
	MH	3000	2300	350	350
	NORMAL	3000	2300	350	350

**Table 2:** Classification results using OCT2017 with a CrossEntropy loss function. Significant values are in [bold].

Dataset	Method	Class	Accuracy	Precision	Recall	Specificity	F1-Score
OCT2017	EfficientNetV2	CNV	0.975	0.913	0.996	0.968	0.953
		DME	0.986	0.996	0.946	0.968	0.970
		DRUSEN	0.977	**1.0**	0.909	0.999	0.952
		NORMAL	0.988	0.953	**1.0**	0.983	0.976
	VIT	CNV	0.950	0.839	0.992	0.937	0.909
		DME	0.975	0.987	0.913	0.996	0.949
		DRUSEN	0.951	0.990	0.814	0.997	0.893
		NORMAL	0.982	0.934	**1.0**	0.977	0.966
	Swin Transformer	CNV	0.995	0.980	**1.0**	0.993	0.990
		DME	**0.999**	**1.0**	0.996	**1.0**	**0.998**
		DRUSEN	0.996	**1.0**	0.983	**1.0**	0.991
		NORMAL	**1.0**	**1.0**	**1.0**	**1.0**	**1.0**
	Swin Transformer V2	CNV	**0.996**	**0.984**	**1.0**	**0.994**	**0.992**
		DME	0.997	**1.0**	**0.988**	**1.0**	0.994
		DRUSEN	**0.999**	**1.0**	**0.996**	**1.0**	**0.998**
		NORMAL	**1.0**	**1.0**	**1.0**	**1.0**	**1.0**

**Table 3:** Classification results using OCT2017 with a PolyLoss function. Significant values are in [bold].

Dataset	Method	Class	Accuracy	Precision	Recall	Specificity	F1-Score
OCT2017	EfficientNetV2	CNV	0.971	0.896	1.0	0.961	0.945
		DME	0.987	**1.0**	0.946	**1.0**	0.972
		DRUSEN	0.976	**1.0**	0.905	**1.0**	0.950
		NORMAL	0.992	0.968	1.0	0.980	0.984
	VIT	CNV	0.952	0.845	0.992	0.939	0.913
		DME	0.978	0.987	0.926	0.996	0.956
		DRUSEN	0.950	0.985	0.814	0.996	0.891
		NORMAL	0.985	0.942	**1.0**	0.979	0.970
	Swin Transformer	CNV	0.997	0.988	**1.0**	**0.996**	**0.994**
		DME	**0.999**	**1.0**	0.996	**1.0**	**0.998**
		DRUSEN	0.998	**1.0**	0.992	**1.0**	**0.996**
		NORMAL	**1.0**	**1.0**	**1.0**	**1.0**	**1.0**
	Ours	CNV	**0.999**	**0.996**	**1.0**	**0.996**	**0.994**
		DME	**0.999**	**1.0**	**1.0**	**1.0**	**0.998**
		DRUSEN	**1.0**	**1.0**	**1.0**	**1.0**	**0.996**
		NORMAL	**1.0**	**1.0**	**1.0**	**1.0**	**1.0**

**Table 4:** Classification results using OCT-C8 with a CrossEntropy loss function. Significant values are in [bold].

Dataset	Method	Class	Accuracy	Precision	Recall	Specificity	F1-Score
OCT-C8	VIT	AMD	**1.0**	**1.0**	**1.0**	**1.0**	**1.0**
		CNV	0.965	0.873	0.846	0.982	0.859
		CSR	0.993	0.958	0.986	0.994	0.972
		DME	0.962	0.901	0.783	0.988	0.838
		DR	0.989	0.954	0.954	0.993	0.954
		DRUSEN	0.943	0.775	0.769	0.968	0.772
		MH	0.991	0.977	0.951	0.997	0.964
		NORMAL	0.959	0.787	0.920	0.964	0.848
	Swin Transformer	AMD	**1.0**	**1.0**	**1.0**	**1.0**	**1.0**
		CNV	**0.988**	0.954	**0.951**	0.993	**0.952**
		CSR	**1.0**	**1.0**	**1.0**	**1.0**	**1.0**
		DME	0.990	0.968	0.957	0.996	0.962
		DR	**1.0**	**1.0**	**1.0**	**1.0**	**1.0**
		DRUSEN	**0.985**	**0.956**	0.937	**0.992**	**0.946**
		MH	**1.0**	**1.0**	**1.0**	**1.0**	**1.0**
		NORMAL	0.987	0.945	**0.977**	0.992	0.961
	Swin Transformer V2	AMD	**1.0**	**1.0**	**1.0**	**1.0**	**1.0**
		CNV	**0.988**	**0.959**	0.940	**0.994**	0.949
		CSR	**1.0**	**1.0**	**1.0**	**1.0**	**1.0**
		DME	**0.992**	**0.974**	**0.963**	**0.996**	**0.968**
		DR	**1.0**	**1.0**	**1.0**	**1.0**	**1.0**
		DRUSEN	**0.985**	0.938	**0.946**	0.991	0.942
		MH	**1.0**	**1.0**	**1.0**	**1.0**	**1.0**
		NORMAL	**0.991**	**0.955**	**0.977**	**0.993**	**0.966**

**Table 5:** Classification results using OCT-C8 with a PolyLoss loss function. Significant values are in [bold].

Dataset	Method	Class	Accuracy	Precision	Recall	Specificity	F1-Score
OCT-C8	VIT	AMD	**1.0**	**1.0**	**1.0**	**1.0**	**1.0**
		CNV	0.967	0.893	0.834	0.986	0.862
		CSR	0.994	0.961	0.991	0.994	0.976
		DME	0.962	0.894	0.794	0.987	0.841
		DR	0.989	0.957	0.957	0.994	0.957
		DRUSEN	0.943	0.772	0.774	0.967	0.773
		MH	0.992	0.985	0.954	0.998	0.969
		NORMAL	0.958	0.781	0.917	0.963	0.844
	Swin Transformer	AMD	**1.0**	**1.0**	**1.0**	**1.0**	**1.0**
		CNV	0.988	0.959	0.943	**0.995**	0.952
		CSR	**1.0**	**1.0**	**1.0**	**1.0**	**1.0**
		DME	0.991	**0.974**	0.957	**0.996**	0.965
		DR	**1.0**	**1.0**	**1.0**	**1.0**	**1.0**
		DRUSEN	**0.988**	0.954	**0.940**	0.993	0.947
		MH	**1.0**	**1.0**	**1.0**	**1.0**	**1.0**
		NORMAL	0.990	0.938	**0.986**	**0.993**	0.961
	Ours	AMD	**1.0**	**1.0**	**1.0**	**1.0**	**1.0**
		CNV	**0.989**	**0.965**	**0.949**	**0.995**	**0.957**
		CSR	**1.0**	**1.0**	**1.0**	**1.0**	**1.0**
		DME	**0.992**	0.963	**0.977**	0.995	**0.970**
		DR	**1.0**	**1.0**	**1.0**	**1.0**	**1.0**
		DRUSEN	**0.988**	**0.965**	0.934	**0.995**	**0.949**
		MH	**1.0**	**1.0**	**1.0**	**1.0**	**1.0**
		NORMAL	**0.991**	**0.948**	0.980	0.992	**0.964**

**Table 6:** Average of experimental results using CrossEntropy and PolyLoss functions on datasets OCT2017 and OCT-C8, respectively. Significant values are in bold.

Dataset	Method	Loss	Accuracy	Precision	Recall	Specificity	F1-Score
OCT2017	EfficientNetV2	CrossEntropy	0.982	0.966	0.963	0.980	0.963
		PolyLoss	0.981	0.966	0.963	0.985	0.963
	VIT	CrossEntropy	0.965	0.938	0.930	0.977	0.917
		PolyLoss	0.966	0.940	0.933	0.978	0.933
	Swin Transformer	CrossEntropy	0.998	0.995	0.995	0.998	0.995
	Paper [30]	PolyLoss	0.998	0.997	0.997	**0.999**	**0.997**
	Swin Transformer V2	CrossEntropy	0.998	0.996	0.996	0.999	0.996
	Ours	PolyLoss	**0.999**	**0.999**	**1.0**	**0.999**	**0.997**
OCT-C8	VIT	CrossEntropy	0.975	0.903	0.901	0.986	0.901
		PolyLoss	0.976	0.905	0.903	0.986	0.903
	Swin Transformer	CrossEntropy	0.994	0.978	0.978	0.997	0.978
	Paper [30]	PolyLoss	0.994	0.978	0.978	**0.997**	0.978
	Swin Transformer V2	CrossEntropy	0.995	0.978	0.978	0.997	0.978
	Ours	PolyLoss	**0.995**	**0.980**	**0.980**	**0.997**	**0.980**

**Table 7:** Experimental results using different models on the OCT2017 and OCT-C8 datasets, respectively. Significant values are indicated in bold.

Dataset	Model	Accuracy	Sensitivity
**OCT2017**	InceptionV3 [39]	0.934	0.978
	MobileNet-v2 [40]	0.985	0.994
	ResNet50-v1 [9]	0.993	0.993
	Joint-Attention-Network ResNet-v1 [41]	0.924	
	Xception [42]	0.997	0.997
	OpticNet-71 [43]	0.998	0.998
	Swin Transformer V1 [30]	0.998	0.998
	**Ours**	**0.999**	**0.999**
**OCT-C8**	VIT	0.975	0.986
	GAN [44]	0.939	
	Swin Transformer	0.994	0.997
	Deep CNN [45]	0.938	
	CenterNet [46]	0.981	
	**Ours**	**0.995**	**0.997**

## Data Availability

Data is contained within the article.

## References

[1] Prem Senthil M., Khadka J., Gilhotra J.S., Simon S., Pesudovs K. (2017). Exploring the quality of life issues in people with retinal diseases: A qualitative study. J. Patient-Rep. Outcomes.

[2] Huang D., Swanson E.A., Lin C.P., Schuman J.S., Stinson W.G., Chang W., Hee M.R., Flotte T., Gregory K., Puliafito C.A. (1991). Optical coherence tomography. Science.

[3] Doi K. (2007). Computer-aided diagnosis in medical imaging: Historical review, current status and future potential. Comput. Med. Imaging Graph. Off. J. Comput. Med. Imaging Soc..

[4] LeCun Y., Bengio Y., Hinton G. (2015). Deep learning. Nature.

[5] Lecun Y., Bottou L., Bengio Y., Haffner P. (1998). Gradient-based learning applied to document recognition. Proc. IEEE.

[6] Krizhevsky A., Sutskever I., Hinton G.E. (2017). ImageNet Classification with Deep Convolutional Neural Networks. Commun. ACM.

[7] Simonyan K., Zisserman A. (2015). Very Deep Convolutional Networks for Large-Scale Image Recognition. arXiv.

[8] Szegedy C., Liu W., Jia Y.Q., Sermanet P., Reed S., Anguelov D., Erhan D., Vanhoucke V., Rabinovich A. Going Deeper with Convolutions. Proceedings of the IEEE Conference on Computer Vision and Pattern Recognition (CVPR).

[9] He K.M., Zhang X.Y., Ren S.Q., Sun J. Deep Residual Learning for Image Recognition. Proceedings of the 2016 IEEE Conference on Computer Vision and Pattern Recognition (CVPR).

[10] Huang G., Liu Z., van der Maaten L., Weinberger K.Q. Densely Connected Convolutional Networks. Proceedings of the 30th IEEE/CVF Conference on Computer Vision and Pattern Recognition (CVPR).

[11] Howard A.G., Zhu M., Chen B., Kalenichenko D., Wang W., Weyand T., Andreetto M., Adam H. (2017). MobileNets: Efficient Convolutional Neural Networks for Mobile Vision Applications. arXiv.

[12] Tan M.X., Le Q.V. EfficientNet: Rethinking Model Scaling for Convolutional Neural Networks. Proceedings of the 36th International Conference on Machine Learning (ICML).

[13] Vaswani A., Shazeer N., Parmar N., Uszkoreit J., Jones L., Gomez A.N., Kaiser L., Polosukhin I. Attention Is All You Need. Proceedings of the 31st Annual Conference on Neural Information Processing Systems (NIPS).

[14] Liu Z., Lin Y.T., Cao Y., Hu H., Wei Y.X., Zhang Z., Lin S., Guo B.N. Swin Transformer: Hierarchical Vision Transformer using Shifted Windows. Proceedings of the 18th IEEE/CVF International Conference on Computer Vision (ICCV), Electr Network.

[15] Dosovitskiy A., Beyer L., Kolesnikov A., Weissenborn D., Zhai X., Unterthiner T., Dehghani M., Minderer M., Heigold G., Gelly S. (2021). An Image is Worth 16x16 Words: Transformers for Image Recognition at Scale. arXiv.

[16] Liu Z., Hu H., Lin Y., Yao Z., Xie Z., Wei Y., Ning J., Cao Y., Zhang Z., Dong L. (2022). Swin Transformer V2: Scaling Up Capacity and Resolution. arXiv.

[17] Leng Z., Tan M., Liu C., Cubuk E.D., Shi X., Cheng S., Anguelov D. (2022). PolyLoss: A Polynomial Expansion Perspective of Classification Loss Functions. arXiv.

[18] Selvaraju R.R., Cogswell M., Das A., Vedantam R., Parikh D., Batra D. (2020). Grad-CAM: Visual Explanations from Deep Networks via Gradient-Based Localization. Int. J. Comput. Vis..

[19] Lee C.S., Baughman D.M., Lee A.Y. (2017). Deep learning is effective for the classification of OCT images of normal versus Age-related Macular Degeneration. Ophthalmology. Retina.

[20] Wang D., Wang L. (2019). On OCT Image Classification via Deep Learning. IEEE Photonics J..

[21] Islam K.T., Wijewickrema S., Leary S.O. Identifying Diabetic Retinopathy from OCT Images using Deep Transfer Learning with Artificial Neural Networks. Proceedings of the 2019 IEEE 32nd International Symposium on Computer-Based Medical Systems (CBMS).

[22] Rong Y.B., Xiang D.H., Zhu W.F., Yu K., Shi F., Fan Z., Chen X.J. (2019). Surrogate-Assisted Retinal OCT Image Classification Based on Convolutional Neural Networks. IEEE J. Biomed. Health Inform..

[23] Fang L.Y., Wang C., Li S.T., Rabbani H., Chen X.D., Liu Z.M. (2019). Attention to Lesion: Lesion-Aware Convolutional Neural Network for Retinal Optical Coherence Tomography Image Classification. IEEE Trans. Med. Imaging.

[24] Singh A., Rasheed M.A., Zelek J., Lakshminarayanan V. Interpretation of deep learning using attributions: Application to ophthalmic diagnosis. Proceedings of the Conference on Applications of Machine Learning, Electr Network.

[25] Wang C., Jin Y., Chen X., Liu Z. (2020). Automatic Classification of Volumetric Optical Coherence Tomography Images via Recurrent Neural Network. Sens. Imaging.

[26] Arefin R., Samad M.D., Akyelken F.A., Davanian A., Soc I.C. Non-transfer Deep Learning of Optical Coherence Tomography for Post-hoc Explanation of Macular Disease Classification. Proceedings of the 9th IEEE International Conference on Healthcare Informatics (IEEE ICHI), Electr Network.

[27] Latha V., Ashok L.R., Sreeni K.G., IEEE Automated Macular Disease Detection using Retinal Optical Coherence Tomography images by Fusion of Deep Learning Networks. Proceedings of the 27th National Conference on Communications (NCC), Electr Network.

[28] Liu X.M., Bai Y.J., Cao J., Yao J.P., Zhang Y., Wang M. (2022). Joint disease classification and lesion segmentation via one-stage attention-based convolutional neural network in OCT images. Biomed. Signal Process. Control.

[29] Esfahani E.N., Daneshmand P.G., Rabbani H., Plonka G. Automatic Classification of Macular Diseases from OCT Images Using CNN Guided with Edge Convolutional Layer. Proceedings of the Annual International Conference of the IEEE Engineering in Medicine and Biology Society.

[30] He J.Z., Wang J.X., Han Z.Y., Ma J., Wang C.J., Qi M. (2023). An interpretable transformer network for the retinal disease classification using optical coherence tomography. Sci. Rep..

[31] Ibrahim M.R., Fathalla K.M., Youssef S.M. (2020). HyCAD-OCT: A Hybrid Computer-Aided Diagnosis of Retinopathy by Optical Coherence Tomography Integrating Machine Learning and Feature Maps Localization. Appl. Sci..

[32] Ai Z., Huang X., Feng J., Wang H., Tao Y., Zeng F.X., Lu Y.P. (2022). FN-OCT: Disease Detection Algorithm for Retinal Optical Coherence Tomography Based on a Fusion Network. Front. Neuroinform..

[33] Arkin E., Yadikar N., Xu X.B., Aysa A., Ubul K. (2023). A survey: Object detection methods from CNN to transformer. Multimed. Tools Appl..

[34] Hendria W.F., Phan Q.T., Adzaka F., Jeong C. (2023). Combining transformer and CNN for object detection in UAV imagery. ICT Express.

[35] Kermany D.S., Goldbaum M., Cai W.J., Valentim C.C.S., Liang H.Y., Baxter S.L., McKeown A., Yang G., Wu X.K., Yan F.B. (2018). Identifying Medical Diagnoses and Treatable Diseases by Image-Based Deep Learning. Cell.

[36] Subramanian M., Shanmugavadivel K., Naren O.S., Premkumar K., Rankish K. Classification of Retinal OCT Images Using Deep Learning. Proceedings of the 2022 International Conference on Computer Communication and Informatics (ICCCI).

[37] Subramanian M., Kumar M.S., Sathishkumar V.E., Prabhu J., Karthick A., Ganesh S.S., Meem M.A. (2022). Diagnosis of Retinal Diseases Based on Bayesian Optimization Deep Learning Network Using Optical Coherence Tomography Images. Comput. Intell. Neurosci..

[38] Tan M.X., Le Q.V. EfficientNetV2: Smaller Models and Faster Training. Proceedings of the International Conference on Machine Learning (ICML), Electr Network.

[39] Szegedy C., Vanhoucke V., Ioffe S., Shlens J., Wojna Z., IEEE Rethinking the Inception Architecture for Computer Vision. Proceedings of the 2016 IEEE Conference on Computer Vision and Pattern Recognition (CVPR).

[40] Sandler M., Howard A., Zhu M.L., Zhmoginov A., Chen L.C. MobileNetV2: Inverted Residuals and Linear Bottlenecks. Proceedings of the 31st IEEE/CVF Conference on Computer Vision and Pattern Recognition (CVPR), Salt Lake City.

[41] Kamran S.A., Tavakkoli A., Zuckerbrod S.L. Improving robustness using joint attention network for detecting retinal degeneration from optical coherence tomography images. Proceedings of the IEEE International Conference on Image Processing (ICIP), Electr Network.

[42] Chollet F. Xception: Deep Learning with Depthwise Separable Convolutions. Proceedings of the 30th IEEE/CVF Conference on Computer Vision and Pattern Recognition (CVPR).

[43] Amit Kamran S., Saha S., Shihab Sabbir A., Tavakkoli A. (2019). Optic-Net: A Novel Convolutional Neural Network for Diagnosis of Retinal Diseases from Optical Tomography Images. arXiv.

[44] Yoo T.K., Choi J.Y., Kim H.K. (2021). Feasibility study to improve deep learning in OCT diagnosis of rare retinal diseases with few-shot classification. Med. Biol. Eng. Comput..

[45] Sathishkumar V.E., Park J., Cho Y. (2020). Seoul bike trip duration prediction using data mining techniques. IET Intell. Transp. Syst..

[46] Nazir T., Nawaz M., Rashid J., Mahum R., Masood M., Mehmood A., Ali F., Kim J., Kwon H.Y., Hussain A. (2021). Detection of Diabetic Eye Disease from Retinal Images Using a Deep Learning based CenterNet Model. Sensors.

